# After delivery: How recipient cells shape the fate and function of transplanted mitochondria

**DOI:** 10.1002/ctm2.70857

**Published:** 2026-10-11

**Authors:** Zhi‐Yuan Zhang, Bo‐Quan Qin, Shi‐Zhou Wu, Hui Zhang

**Affiliations:** ^1^ Department of Orthopedics Orthopedic Research Institute West China Hospital Sichuan University Chengdu People's Republic of China

**Keywords:** endosomal escape, heteroplasmy, innate immunity, mitochondrial fusion, mitochondrial transplantation, mitophagy, translational medicine

## Abstract

**Background:**

Mitochondrial transplantation is built on a simple idea: failing cells might be rescued by healthy mitochondria. The approach has entered early clinical testing, and encapsulation and cell‐type targeting have improved uptake in selected systems. Better delivery, however, does not explain the therapy.

**Main body:**

Internalised donor mitochondria encounter an established network, quality‐control machinery and sensors for mitochondrial material. Donor material may remain in the endolysosomal system or reach the cytosol. Cytosolic fates include network integration, clearance and a candidate functional contribution without fusion. Intracellular processing may also generate bioactive cargo. These fates imply different mechanisms and evidence requirements. This Review examines how recipient cells shape the contribution of internalised mitochondria, while recognising extracellular routes to benefit. We distinguish direct transplantation evidence from mechanistic support drawn from endogenous mitochondrial biology and natural intercellular transfer. The required donor fate and duration depend on the therapeutic task. Transient rescue may rely on short‐term donor function or productive processing in specific models; sustained support may depend on continued donor function, recipient adaptation or redosing. Durable mitochondrial DNA (mtDNA) correction requires donor‐mtDNA retention and replication.

**Conclusions:**

Linking these tasks to product design, safety assessment and clinical endpoints shifts the question from how many mitochondria enter cells to what they accomplish after arrival.

**Key points:**

- Recipient‐cell handling shapes what transplanted mitochondria do after uptake.
- Uptake, structural integration and donor mitochondrial DNA retention require distinct evidence.
- Transient rescue, sustained support and durable mitochondrial DNA correction impose different requirements.
- Therapeutic goals should guide donor selection, product testing and clinical endpoints.

## Graphical abstract

Recipient‐cell handling shapes how transplanted mitochondria contribute after delivery, linking donor competence, recipient state and exposure duration to intracellular fate. The framework matches transient rescue, sustained functional support and durable mitochondrial DNA correction to the evidence and product attributes required for each therapeutic task.

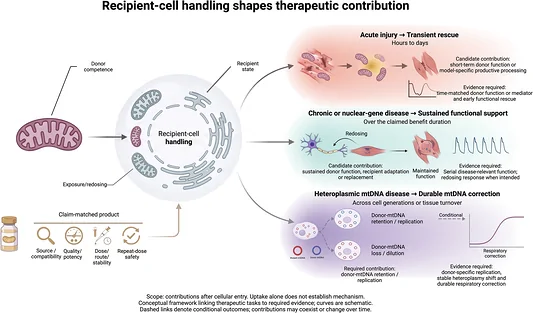

**Abbreviations**
ATP: adenosine triphosphate
BNIP3: BCL2‐interacting protein 3
cGAMP: cyclic GMP–AMP
cGAS: cyclic GMP–AMP synthase
DRP1: dynamin‐related protein 1
ESCRT: endosomal sorting complexes required for transport
FUNDC1: FUN14 domain‐containing protein 1
GABARAP: GABA type A receptor‐associated protein
IRF3: interferon regulatory factor 3
LC3: microtubule‐associated protein 1 light chain 3
MFN1/2: mitofusins 1 and 2
mtDNA: mitochondrial DNA
NDP52: nuclear dot protein 52
NIX: BCL2‐interacting protein 3‐like
NLRP3: NLR family pyrin domain containing 3
OMA1: OMA1 zinc metallopeptidase
OPA1: optic atrophy 1
OPTN: optineurin
PINK1: PTEN‐induced kinase 1
STING: stimulator of interferon genes
TBK1: TANK‐binding kinase 1
TLR9: Toll‐like receptor 9
ULK1: unc‐51‐like autophagy‐activating kinase 1

## 1 INTRODUCTION

Mitochondrial transplantation aims to rescue damaged cells with functional mitochondria. Early studies delivered isolated mitochondria to ischaemic myocardium during reperfusion.^1^, ^2^ Clinical testing now includes autologous transplantation for paediatric cardiac ischaemia–reperfusion injury^3^ and a first‐in‐human phase 1 trial in acute cerebral ischaemia.^4^

Delivery remains a practical obstacle. Quantitative cellular studies show that uptake can be inefficient and does not ensure productive intracellular delivery.^5^ Encapsulation in erythrocyte‐membrane‐derived vesicles increased uptake‐positive cells from below 5% to about 80% in the reported system.^6^ MitoCatch enables tunable attachment to selected recipient cells.^7^

Better delivery, however, does not explain the therapy. Internalised donors encounter an established network, quality‐control machinery and sensors for mitochondrial material. They may integrate structurally or support function while remaining unfused. Benefit could also arise from released mitochondrial DNA (mtDNA), metabolites or peptides, or recipient responses to damage‐associated signals.^8^, ^9^, ^10^, ^11^, ^12^ Similar tissue‐level improvements could therefore reflect different mechanisms.

A recent synthesis reviewed mitochondrial‐transfer technologies and therapeutic applications,^13^ and consensus recommendations established nomenclature and characterisation standards.^14^ We ask how recipient‐cell handling shapes transplanted mitochondria's contribution, and what evidence distinguishes the possible mechanisms.

Recipient cells handle donor material through systems that maintain endogenous mitochondria and process incoming cargo. Endolysosomal pathways sort internalised material.^15^, ^16^ Compatible donors may fuse with the resident network,^17^, ^18^ while mitophagy and genome‐level selection shape what is retained or removed.^19^, ^20^, ^21^ Innate sensors can recognise mitochondrial components as bacterial‐like or damage‐associated signals.^11^, ^22^, ^23^, ^24^

We follow delivery through recipient‐cell handling to therapeutic contribution. Transient rescue, sustained support and durable mtDNA correction demand different donor functions and persistence, guiding measurement and product design.

### 1.1 Review approach and evidence framework

This narrative Review prioritises primary transplantation studies of recipient‐cell handling and therapeutic outcomes. Endogenous mitochondrial biology and natural intercellular transfer provide mechanistic context. Claims were checked against accessible full texts or, where unavailable, verifiable abstract information. We distinguish direct transplantation evidence, mechanistic support from adjacent fields and hypotheses requiring testing.

Four targeted Europe PMC searches on 13 September 2026 covered reports first published from 1 January to that date. Mitochondrial transplantation or transfer terms were combined with donor source, product integrity, extracellular mechanisms, clinical studies and safety terms. Crossref verified bibliographic metadata.

## 2 HOW DELIVERY SHAPES WHAT HAPPENS NEXT

Delivery sets the starting conditions. Endocytic uptake places donor material behind an endolysosomal membrane; direct cytosolic transfer bypasses it. Local and vascular administration expose different recipient populations.

### 2.1 Route and bulk delivery

Direct injection or vascular infusion exposes tissue to isolated mitochondria.^25^, ^26^ Autologous transplantation studies in paediatric cardiac ischaemia–reperfusion injury and acute cerebral ischaemia illustrate source‐tissue accessibility and provide preliminary short‐term safety observations.^3^, ^4^ Preclinical injury models also show functional benefit.^25^, ^27^, ^28^, ^29^ Yet uneven, inefficient uptake leaves tissue exposure better characterised than individual organelles’ post‐entry fate.^5^

Direct injection concentrates local exposure; vascular infusion exposes donors to blood and endothelium before parenchymal delivery. In experimental Parkinson's disease, intranasally delivered mitochondria have been detected along olfactory and neuronal routes.^30^ Intravenous delivery has separately been associated with neuronal protection, reduced neuroinflammation and behavioural improvement.^31^ Cell‐resolved measurements must identify recipients and subsequent donor fate.

### 2.2 Encapsulation

The reported erythrocyte‐membrane‐derived vesicle system increased the proportion of cells positive for donor mitochondria from below 5% to approximately 80%. In selected cellular systems, encapsulated mitochondria associated with the endogenous network, donor‐derived mtDNA remained detectable for at least 7 days, and bioenergetic defects were corrected. Functional rescue was also reported in models of mtDNA‐depletion syndrome, Leigh syndrome and Parkinson's disease.^6^

The advantage could reflect preserved donor membrane potential during extracellular transit, altered internalisation, reduced lysosomal degradation or a larger intact dose reaching receptive cells. Time‐resolved in vivo tracking should combine donor‐specific respiratory measurements with perturbation of recipient fusion or clearance. Improved delivery establishes neither an intracellular mechanism nor durable donor‐genome maintenance in unselected tissues. Patient‐derived cell and animal findings do not establish clinical benefit. Independent replication, clinically relevant dosing and long‐term donor‐specific tracking remain priorities.

### 2.3 Cell‐type targeting and surface engineering

Targeting determines which cells encounter donor mitochondria. MitoCatch uses cell‐surface, mitochondrion‐displayed or bispecific binders to target defined recipients. In tested systems, donors were internalised and exposed to the cytosol; transport, fusion and fission were observed. Targeted transplantation protected damaged neurons in vitro and after neuronal injury in mice.^7^ Whether protection requires fusion, and how long donor mtDNA persists, remain unresolved.

Surface engineering has a narrower evidence base. Peptides targeting vascular cell adhesion molecule 1 or collagen increased mitochondrial association, uptake and retention in injured endothelial cells, alongside improved mitochondrial function.^32^ Improvement alone cannot separate donor activity from endogenous‐network responses. Cytosolic‐access reporters, donor‐specific genome tracking and time‐resolved imaging are needed.

Recipient identity is part of the mechanism, not merely a targeting parameter. Cardiomyocytes require energetic rescue and fusion competence within dense, largely post‐mitotic networks. Neuronal polarity imposes transport and compartmental constraints; endothelial and immune cells connect endolysosomal handling to vascular or innate responses. Proliferative state, network integrity, endolysosomal activity and mitophagy capacity can shape donor fate.^8^, ^10^, ^30^, ^31^ Matched transplantation studies across lineages remain limited. Natural intercellular transfer supports cellular context's importance,^33^, ^34^, ^35^ but may bypass barriers faced by isolated organelles.

### 2.4 Ex vivo transfer platforms

Selected ex vivo transfer platforms bypass the endosomal barrier by placing donor mitochondria directly in the cytosol. The photothermal nanoblade transiently opens the plasma membrane,^36^ whereas MitoPunch drives mitochondrial suspensions through transient membrane disruptions.^37^ In mtDNA‐deficient rho‐zero recipient cells, these approaches generated clones that retained donor mtDNA and recovered respiration.^36^, ^37^ Respiratory rescue has also been reported after simple co‐incubation, which does not establish direct cytosolic delivery.^38^

Mechanical entry, rho‐zero recipients and metabolic or clonal selection constrain extrapolation to intact tissues.^36^, ^37^ These systems establish possible outcomes, not their frequency in unselected, mitochondrially intact cells. MitoCeption uses centrifugation to increase organelle–cell contact without guaranteeing cytosolic placement.^39^ Platform comparisons must specify the barrier removed and fate measured.

### 2.5 Separating delivery from mechanism

These platforms alter tissue exposure, donor protection, recipient selection or access to the cytosol. Some mitochondrial preparations can act before entry: extracellular nanoscale mitochondria have been reported to scavenge reactive oxygen species without cellular uptake.^40^ Such effects lie outside the post‐entry sequence examined here.

Uptake denotes internalisation, usually into a membrane‐bound compartment. Cytosolic access denotes exposure of an intact donor organelle or donor material to recipient cytosol. Structural integration requires donor–recipient matrix mixing or resolved membrane and matrix continuity. Persistence denotes retention of an intact organelle or donor mtDNA over a defined interval. Evidence for one event does not establish the next; Table 1 specifies minimum readout**s**.

**TABLE 1:** Readouts that separate delivery, fate and mechanism. Each question is paired with the minimum readout, the conclusion it supports and the limits of that inference.

Question	Minimum readout	What it can show	What it cannot prove alone
Was donor material delivered to tissue or associated with cells?	Tissue retention; cell‐resolved imaging or flow cytometry with appropriate controls	Tissue exposure or cell association, according to the readout	Cytosolic access, viability or integration
Did they reach the cytosol?	Cytosolic‐access reporters and compartment‐resolved imaging; galectin or rupture markers as supporting evidence	Exposure of an intact donor organelle or donor material to the cytosol	Organelle integrity, structural integration or durable function
Did they integrate?	Donor–recipient matrix mixing or resolved membrane and matrix continuity; colocalisation and MFN1/2‐ or OPA1‐dependence are supportive	Structural integration into the recipient network	Donor‐mtDNA persistence, functional necessity or therapeutic benefit
Did donor mtDNA persist?	Serial donor‐specific mtDNA measurements; replication assays and heteroplasmy tracking when replication or correction is claimed	Donor‐mtDNA retention over the measured interval; replication or heteroplasmy change when directly measured	Replication from sequence detection alone; durable correction, respiratory contribution or clinical benefit without corresponding evidence
Were they cleared or processed?	Donor‐resolved degradation and flux assays; PINK1–Parkin recruitment and lysosomal routing indicate engagement	Quality‐control engagement or completed clearance, according to the readout	Productive processing without functional and perturbation evidence
Did they contribute respiration?	Donor‐resolved functional assays; bulk oxygen consumption, ATP or membrane potential require donor‐attribution controls	Respiratory contribution attributable to donor mitochondria when the source is resolved	Fusion dependence or persistence; bulk bioenergetic changes alone cannot identify donor contribution
How long does benefit last?	Serial functional measurements, donor persistence and, for mtDNA correction, replication and heteroplasmy dynamics	Alignment with transient rescue, sustained functional support or durable mtDNA correction	The active mechanism from duration alone
Which mechanism explains benefit?	Targeted perturbation with controls for delivery, donor quality and recipient basal function; rescue or orthogonal tests	Support for the necessity of a specific post‐entry event, subject to controls	Necessity from inhibition alone; universality across diseases or cell types

Abbreviations: ATP, adenosine triphosphate; MFN1/2, mitofusins 1 and 2; mtDNA, mitochondrial DNA; OPA1, optic atrophy 1; PINK1, PTEN‐induced kinase 1.

## 3 AFTER ENTRY: THE FATE OF A TRANSPLANTED MITOCHONDRION

Uptake is not fate. Within an endosome or lysosome, a donor mitochondrion remains separated from the recipient network and may be retained, processed or degraded. Cytosolic donor material instead encounters the machinery that moves, remodels, fuses and removes mitochondria.

Figure 1 maps the branching fates: compartmental retention, cytosolic access, integration, remodelling, clearance and candidate non‐fused function. These may coexist. Selected ex vivo methods bypass endolysosomal barriers; transplantation studies demonstrate access and integration in selected systems. Escape mechanisms, fate frequencies and persistence durations remain less resolved.

**FIGURE 1:**
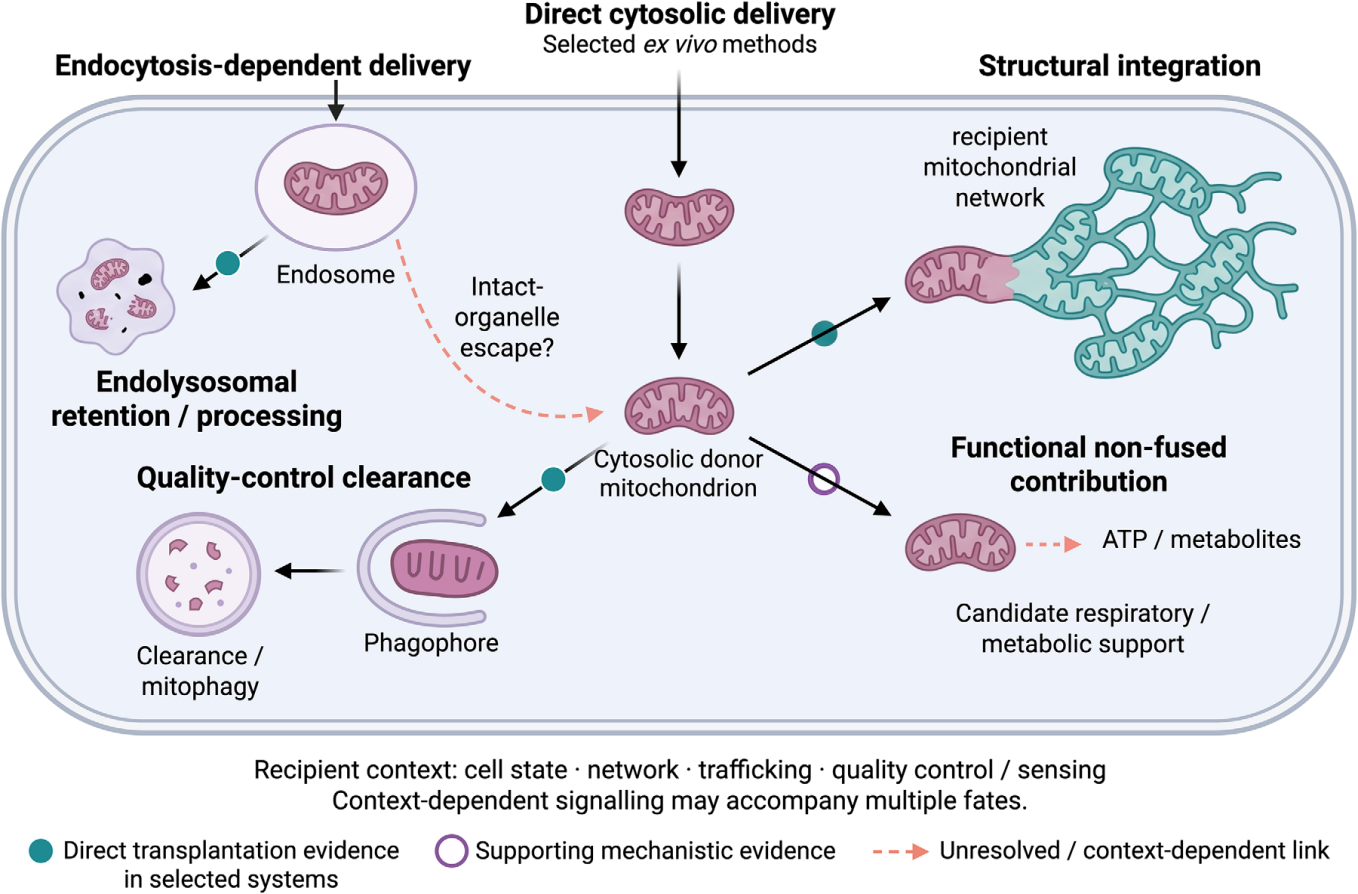
Recipient‐cell control of post‐entry mitochondrial fate. Endocytosed donor mitochondria may remain in the endolysosomal system or undergo processing. How an intact organelle crosses the endolysosomal membrane remains unresolved; selected ex vivo methods provide direct cytosolic delivery. Cytosolic donors may integrate with the recipient network, undergo mitophagic clearance or provide support without fusion (functional non‐fused contribution). The last route denotes candidate respiratory or metabolic support, with a conditional link to ATP and metabolites. Recipient‐cell state, network properties, trafficking, quality control and sensing can shape these fates; signalling may accompany several routes. The routes can coexist or occur sequentially, without forming obligatory stages of a single pathway. Filled teal circles denote direct transplantation evidence in selected systems; hollow purple circles denote mechanistic support; coral dashed arrows denote unresolved or context‐dependent links. Solid arrows show process direction, not evidence strength. ATP, adenosine triphosphate.

Donor condition and recipient state jointly shape these fates. Membrane potential is one indicator of donor quality: polarised mitochondria are more likely to remain fusion‐competent, whereas depolarisation favours removal.^19^, ^41^, ^42^ Isolation injury, lysosomal activity, mitonuclear compatibility, donor source and recipient‐network state also influence what follows entry.

### 3.1 Escape after endocytic uptake: the first unresolved step

Endosomal escape becomes a bottleneck when uptake places donor mitochondria inside a membrane‐bound compartment.^16^ The compartment separates the donor from cytosolic machinery for movement, fusion and quality control.

Transplantation studies demonstrate actin‐dependent endocytosis and macropinocytosis‐like entry.^15^, ^16^ Cargo may remain in the endolysosomal system or be degraded. Intact‐donor escape requires crossing the compartment boundary, but the molecular events remain unresolved.

Photothermal nanoblade and MitoPunch place donor mitochondria directly in the cytosol through transient plasma‐membrane disruption.^36^, ^37^ Fusogenic lipid capsules instead use carrier–cell membrane fusion to deliver mitochondria to chondrocytes.^43^ Natural transfer through cellular contacts or conduits can also bypass parts of the endosomal route faced by isolated organelles.^33^, ^35^, ^44^, ^45^ Bypassing this barrier does not explain how an endocytosed, intact mitochondrion crosses it.

Endolysosomal damage need not lead to escape. Limited injury can recruit the endosomal sorting complexes required for transport (ESCRT) for repair.^46^ Exposed luminal glycans recruit galectins and may initiate autophagic processing or lysophagy.^47^ These responses could reseal or degrade the compartment, or accompany a productive breach. Figure 2A marks this mechanistic support; transplantation‐specific roles remain untested.

**FIGURE 2:**
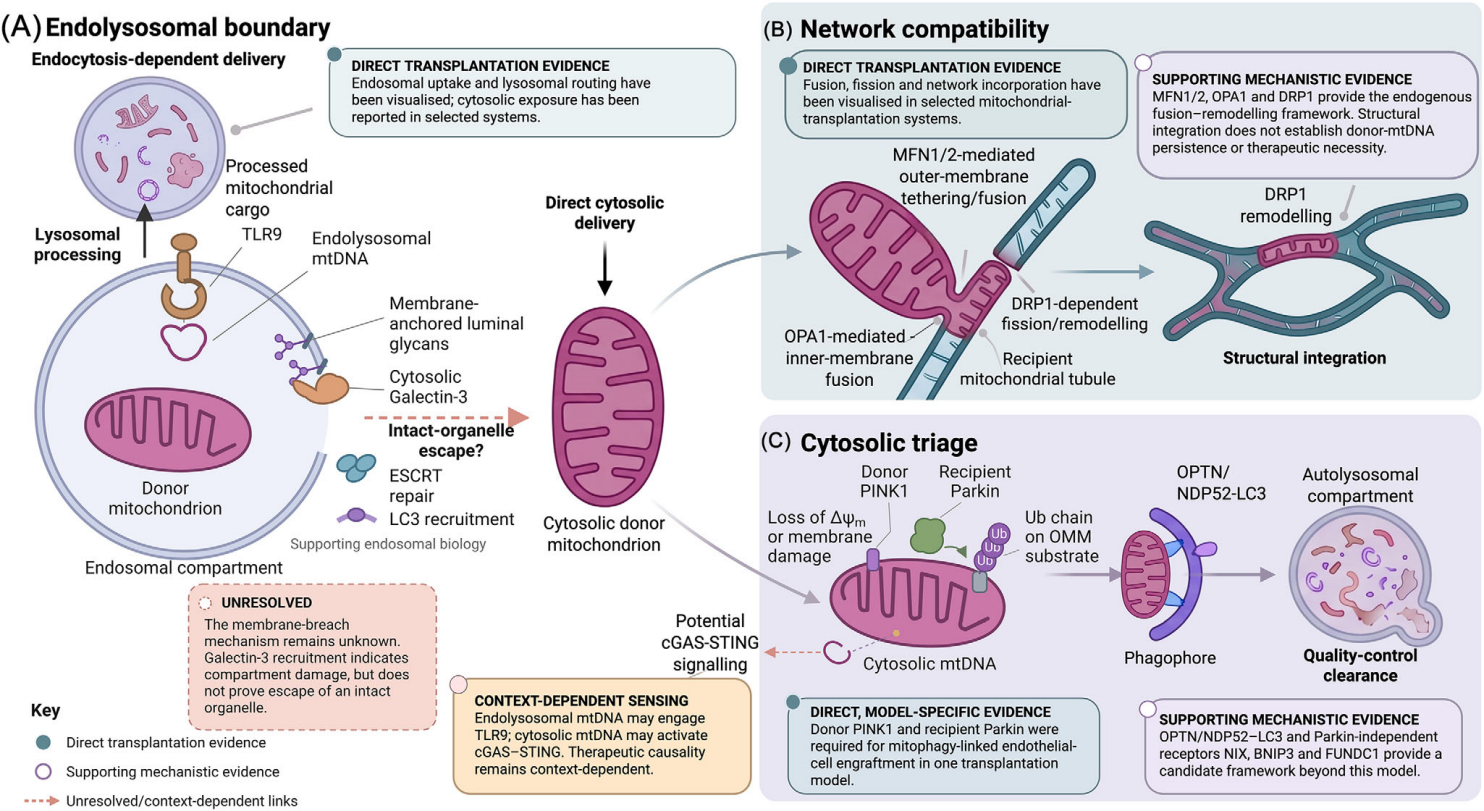
Recipient‐cell checkpoints and evidence hierarchy governing the post‐entry fate of transplanted mitochondria. (A) Endocytosed mitochondria may undergo lysosomal processing or reach the cytosol; direct cytosolic delivery bypasses the endosomal barrier. Compartment damage exposes membrane‐anchored luminal glycans to cytosolic galectin‐3. Recruitment of galectin‐3 marks damage, not escape of an intact organelle. ESCRT‐mediated repair and LC3 recruitment provide supporting endosomal mechanisms; the membrane‐breach process remains unresolved. Endolysosomal mtDNA may engage TLR9 and cytosolic mtDNA may activate cGAS–STING. Their contribution to benefit depends on context and requires causal testing. (B) MFN1/2 mediate outer‐membrane tethering and fusion, OPA1 mediates inner‐membrane fusion, and DRP1 supports fission and remodelling in endogenous mitochondria. Fusion, fission and network incorporation have been visualised in selected transplantation systems. The molecular requirements remain incompletely tested in transplantation, and structural integration alone establishes neither donor‐mtDNA persistence nor therapeutic necessity. (C) Membrane depolarisation or damage can prompt cytosolic quality‐control triage, without imposing a binary fate rule. The candidate PINK1–Parkin pathway shows Parkin‐dependent ubiquitin chains on outer‐mitochondrial‐membrane substrates, OPTN/NDP52–LC3‐associated phagophore recruitment and autolysosomal clearance. Donor PINK1 and recipient Parkin were required for mitophagy‐linked endothelial‐cell engraftment in one transplantation model. Support for the broader pathway, including OPTN/NDP52–LC3 and the Parkin‐independent receptors NIX, BNIP3 and FUNDC1, comes from endogenous quality‐control biology. Solid arrows show process direction; coral dashed arrows denote unresolved or context‐dependent links. Filled teal circles identify direct transplantation evidence, including model‐specific evidence; hollow purple circles identify mechanistic support. BNIP3, BCL2‐interacting protein 3; cGAS, cyclic GMP–AMP synthase; DRP1, dynamin‐related protein 1; ESCRT, endosomal sorting complexes required for transport; FUNDC1, FUN14 domain‐containing protein 1; LC3, microtubule‐associated protein 1 light chain 3; MFN1/2, mitofusins 1 and 2; mtDNA, mitochondrial DNA; NDP52, nuclear dot protein 52; NIX, BCL2‐interacting protein 3‐like; OMM, outer mitochondrial membrane; OPA1, optic atrophy 1; OPTN, optineurin; PINK1, PTEN‐induced kinase 1; STING, stimulator of interferon genes; TLR9, Toll‐like receptor 9; Ub, ubiquitin; Δψm, mitochondrial membrane potential.

Viral and lipid‐nanoparticle escape are imperfect analogies for moving a 0.5–1 µm organelle across a membrane. Galectin surveillance, microtubule‐associated protein 1 light chain 3 (LC3)‐associated processing and ESCRT repair inform studies of damaged compartments.^46^, ^47^, ^48^ None have been shown to release an intact, viable donor mitochondrion. The decisive observation would link damage to that organelle's membrane crossing.

Cowan et al.^8^ traced divergent donor fates in human induced pluripotent stem cell‐derived cardiomyocytes and primary cardiac fibroblasts. Donor mitochondria passed through endosomal and lysosomal compartments; a subset subsequently reached and fused with the recipient network, whereas others entered degradative pathways. The membrane‐breach event itself remained unresolved.

Encapsulation and MitoCatch provide different levels of evidence for cytosolic access. Du et al.^6^ observed dynamic donor mitochondria, reduced lysosomal colocalisation and subsequent donor–host network fusion after encapsulated delivery. They also reported donor‐mtDNA detection in selected cellular systems and in mouse tissues at 48 h, alongside functional rescue in patient‐derived cells and animal models. Tissue‐level mtDNA detection does not establish the duration of intact donor‐organelle survival. MitoCatch linked targeted uptake to cytosolic access and network dynamics over an acute interval.^7^ How often escape succeeds and which machinery enables it remain unresolved. For these platforms, persistence across cell division or tissue turnover still requires direct testing.

A quantitative study separated events often grouped as successful delivery. Mitochondrially targeted split‐luciferase complementation was consistent with donor–recipient interaction and content mixing. Approximately 9% of internalised mitochondria colocalised with galectin‐3 puncta.^5^ Galectin recruitment marks compartment damage, not completed intact‐organelle escape. Uptake, membrane damage, cytosolic exposure and integration require distinct evidence.

Escape experiments should track donor material across compartments and time. Compartment‐resolved imaging and cytosolic‐access reporters can establish exit, followed by assessment of donor integrity, network behaviour and function. Perturbing endosomal maturation, acidification, ESCRT or galectin–LC3 responses requires early‐uptake controls. Comparing endocytic and direct cytosolic delivery at matched donor exposure and viability would isolate escape's contribution to subsequent fate.

### 3.2 Integration: joining the host network

Cytosolic access brings donors within reach of the recipient network. Structural integration has been directly observed,^8^ whereas donor‐mtDNA retention is a distinct genomic outcome. Neither alone establishes fusion as the cause of benefit.

Fusion requires coordinated donor and recipient machinery. Mitofusins 1 and 2 (MFN1 and MFN2) mediate outer‐membrane tethering and fusion,^17^ whereas optic atrophy 1 (OPA1) completes inner‐membrane fusion and maintains cristae organisation.^18^, ^49^ Dynamin‐related protein 1 (DRP1) participates in network remodelling and the separation of damaged mitochondrial regions.^41^, ^50^ Competent donor fusion machinery must therefore encounter a recipient network capable of accepting it. Figure 2B places observed integration within this endogenous fusion–remodelling framework.

Membrane potential links fusion competence to quality control. In endogenous mitochondria, depolarisation promotes OPA1 processing by OMA1 zinc metallopeptidase (OMA1), restricts inner‐membrane fusion and favours PTEN‐induced kinase 1 (PINK1)–Parkin‐mediated removal.^19^, ^41^, ^42^ FluidFM and targeted‐delivery studies found that intact donors often entered fusion–fission dynamics, whereas injured or purification‐damaged organelles were more likely to fragment or degrade.^7^, ^51^ No single membrane‐potential threshold spans donor sources, delivery routes and recipient types. Isolation injury, oxidative damage, mitonuclear compatibility and recipient‐network state also shape whether integration remains possible.

Transplantation studies capture network behaviour and genomic outcomes in selected systems. FluidFM demonstrated rapid fusion and donor‐mtDNA replication across recipient generations^51^; MitoCatch linked cytosolic access to fusion and fission.^7^ Encapsulation produced genetic complementation and heteroplasmy changes.^6^

Fusion is only one possible route to donor function. Functional non‐fused contribution describes a distinct candidate fate: a physically separate donor may provide respiratory or metabolic support, at least transiently. Ali Pour et al. found higher whole‐cell basal respiration and adenosine triphosphate (ATP) production in H9c2 cardiomyoblasts two days after transplantation.^9^ These measurements establish early bioenergetic benefit but leave donor fusion status unresolved. By contrast, a 2026 retinal ganglion‐cell study found attenuated transplantation benefit under fusion inhibition, supporting a contribution from fusion in that model.^52^ Genetic complementation provides evidence of genomic and functional contribution in selected systems,^53^ while natural intercellular‐transfer studies provide complementary evidence for respiratory rescue.^33^, ^54^ Fusion dependence must therefore be tested in the relevant recipient context, rather than inferred from recovery alone.

Recipient‐network quality can alter fusion's value. Content mixing may complement a metabolic defect, whereas MFN2 or OPA1 abnormalities can constrain integration.^55^, ^56^ Depolarised or oxidatively damaged networks may quarantine, clear or dilute healthy donors, exposing them to recipient pathology. Selective fusion can limit harmful mixing,^41^ but extensive damage may overwhelm this safeguard. Integration could become counterproductive, although direct evidence of such transplantation‐associated harm is lacking.

Testing fusion dependence requires perturbing recipient MFN1, MFN2 or OPA1 while matching early uptake, cytosolic access and donor viability. Matrix‐mixing reporters and donor‐specific sequencing can track structural integration and donor‐mtDNA persistence alongside respiration and disease‐relevant outcomes. Loss of durable rescue under verified fusion blockade would strengthen the case for fusion dependence if recipient basal respiration and network integrity were preserved. Genetic rescue or orthogonal perturbations would further support that inference. Benefit that persists under verified blockade would instead favour non‐fused function or signalling. Comparisons between healthy and fusion‐deficient recipients would test how this requirement depends on recipient‐network state; matched comparisons between proliferating and post‐mitotic recipients would test its dependence on proliferative state.

Structural integration is a possible donor fate, not an obligatory endpoint. Its value depends on donor competence, recipient state, disease mechanism and required correction duration.

### 3.3 Clearance and persistence over time

After cytosolic access or integration, donors enter a population under continuous remodelling, selection and renewal. They may be retained, diluted, dismantled or degraded. Donor mtDNA or a recipient response may outlast the intact organelle. What remains, and in what form, matters more than detection alone.

Degradation before cytosolic access is endolysosomal processing, distinct from mitophagy of a cytosolic donor. Cytosolic donors encounter endogenous quality‐control machinery. Fission can separate poorly polarised regions, while selective fusion excludes damaged material and favours autophagic removal.^19^, ^41^, ^42^ Defects that impair integration may also accelerate clearance.

Depolarised cytosolic mitochondria can stabilise PINK1 and initiate phospho‐ubiquitin signalling and Parkin‐dependent ubiquitination. The autophagy receptors optineurin (OPTN) and nuclear dot protein 52 (NDP52) engage downstream machinery, including TANK‐binding kinase 1 (TBK1), unc‐51‐like autophagy‐activating kinase 1 (ULK1), LC3 and GABA type A receptor‐associated protein (GABARAP).^20^, ^57^ BCL2‐interacting protein 3‐like (NIX), BCL2‐interacting protein 3 (BNIP3) and FUN14 domain‐containing protein 1 (FUNDC1) provide context‐dependent Parkin‐independent routes.^58^, ^59^, ^60^ Established chiefly in endogenous mitochondria, this circuitry supplies a candidate framework for donor‐organelle triage (Figure 2C).

Transplantation studies connect clearance to distinct outcomes. FluidFM transfer revealed that most cytosolic donors entered fusion dynamics, whereas a subset fragmented or degraded; donor mtDNA could propagate at low abundance.^51^ In endothelial cells, donor PINK1 and recipient Parkin were required for mitophagy and enhanced vascular engraftment despite the absence of stable structural integration.^10^ Productive processing thus has causal support in one endothelial model. A separate stroke study combined coated mitochondria with resveratrol and reported restored autophagic flux and reduced neuron–microglia inflammatory signalling.^61^

Organelle, genome and label require separate tracking. Tissue fluorescence or iron retention can outlast donor rupture, lysosomal processing and debris uptake.^5^ Donor‐specific mtDNA identifies retained genetic material; replication more strongly supports genome maintenance.^51^ Stable heteroplasmy shifts and respiratory correction assess genetic and functional outcomes. Integration, donor‐genome persistence and tracer persistence have distinct lifetimes and are not interchangeable.

Required persistence follows the therapeutic claim. Brief donor survival may support acute metabolism or initiate longer‐lived recipient responses. Durable heteroplasmy correction requires donor genomes to persist, replicate and withstand cell division or tissue turnover.^21^, ^53^ Genome loss limits this mechanism. Mitophagy inhibition that prolongs retention but reduces benefit would implicate productive processing; improved rescue through preserved donor respiration would instead implicate clearance as a constraint.

Donor source and compatibility require assessment during acute and sustained exposure, alongside quality, dose and recipient background. Autologous donors reduce sequence disparity; allogeneic or xenogeneic donors introduce distinct mtDNA haplotypes, encoded peptides and other components.^62^, ^63^, ^64^, ^65^ Retention and redosing add questions of genome maintenance, mitonuclear compatibility and cumulative immunity. Box 1 separates source and exposure from preparation quality.

Persistence is not inherently beneficial. Cancer‐derived mitochondria can enter tumour‐infiltrating T cells, evade mitophagy and impair function.^66^ Retained donor material may restore a missing function but can also carry pathogenic genomes, maladaptive metabolites or inhibitory signals. The relevant endpoint is disease‐specific benefit without new liability.

Donors matched for early cytosolic access and membrane potential can be followed over time with organelle pulse–chase, lysosome‐sensitive mitophagy reporters and donor‐specific mtDNA assays. Perturbing donor PINK1 or recipient PRKN (encoding Parkin), followed by rescue, can test how clearance affects respiration or the disease phenotype.^10^ Comparisons of donor source should control for differences in organelle quality and initial delivery.

**Box 1 How donor source, product state and exposure pattern shape risk**

Mitochondria are unusual grafts. They lack the donor major histocompatibility complex class I display of intact nucleated cells, making cellular allografts an imperfect analogy. Free mtDNA, N‐formyl peptides, cardiolipin, membrane disruption and other preparation‐associated material can engage innate immune pathways.^11^, ^22^, ^24^, ^67^, ^68^, ^69^ Donor source may modify these responses and must be assessed alongside composition, integrity, dose and route, without assuming that one variable dominates.

Source compatibility matters across exposure durations. Repeated dosing adds questions about cumulative and adaptive responses. Donor‐derived peptides and mtDNA haplotypes can support adaptive recognition in cellular and animal settings,^63^, ^64^ although one mitochondrial‐injection study found no clear alloreactivity under the conditions tested.^65^ PN‐101 provides early human experience with single‐dose allogeneic transplantation,^70^ leaving safety under repeated or prolonged exposure unresolved. Donor identity and product integrity remain separate variables. Autologous tissue may carry disease‐relevant or tissue‐specific heteroplasmy,^71^ whereas allogeneic or engineered sources add questions of genotype and mitonuclear compatibility.^62^ All sources require matched assessment of organelle integrity, accompanying cargo and intended exposure duration.

Abbreviation: mtDNA, mitochondrial DNA.

### 3.4 Organelle or signal? A question of attribution

Integration, persistence, candidate non‐fused function and productive processing may yield similar benefits and coexist with bioactive‐cargo release. Attribution requires identifying which donor state sustains benefit, and when.

Rapid cardiac responses keep transient mechanisms in view. Experimental myocardial benefit can appear within minutes,^2^, ^26^ without establishing stable integration as its cause. Cardiac dose–response saturation^25^, ^26^ and short‐lived bioenergetic improvement after non‐autologous transfer in cultured cardiomyoblasts^9^ also permit transient contributions. These findings identify no mediator; early recovery may involve transient donor function or signalling beyond integrated respiration.

The endothelial mitophagy result poses a specific attribution problem: processing was required for enhanced vascular engraftment, but its downstream mediator remains unresolved.^10^ Osteocyte mitochondria increased cytosolic mtDNA and activated cyclic GMP–AMP synthase (cGAS)–stimulator of interferon genes (STING) signalling in tumour cells. Isolated‐mitochondria transfer reproduced key features of that response.^12^ By contrast, transplantation into inflamed synoviocytes and arthritic mice restored mitochondrial and lysosomal function while suppressing abnormal cGAS–STING activity.^72^ Donor material can therefore activate sensing or reduce it by restoring recipient‐cell homeostasis.

Compartment and exposure shape immune recognition. Endolysosomal mtDNA can engage Toll‐like receptor 9 (TLR9),^67^, ^73^ whereas cytosolic mtDNA can activate the cGAS–cGAMP–STING–TBK1–IRF3 axis (cGAMP, cyclic GMP–AMP; IRF3, interferon regulatory factor 3).^68^, ^74^ Oxidised mtDNA, ATP, N‐formyl peptides and cardiolipin can engage NLR family pyrin domain containing 3 (NLRP3), formyl‐peptide receptors and related innate pathways.^11^, ^24^, ^69^ Innate responses can emerge rapidly; adaptive recognition requires separate assessment across exposure settings.^63^, ^64^, ^65^ Figure 2A,C distinguishes these sensing routes from evidence that they mediate therapeutic benefit.

Organelle function and signalling can coexist. A donor could first fuse and contribute respiration, then release bioactive material during dismantling. In natural immune‐to‐tumour transfer, acquired mitochondria fused before donor mtDNA leaked and activated cGAS–STING.^75^ This natural‐transfer sequence, not an isolated‐transplantation result, shows how donor output can change. Each stage must be linked to the disease phenotype.

A live‐versus‐dead donor comparison cannot by itself distinguish these mechanisms. Freezing, disruption and heat inactivation alter respiration, membrane integrity, metabolites and danger‐associated cargo together. In oxidatively injured cultured astrocytes, lipid‐encapsulated mitochondria‐associated messenger RNAs restored ATP through cytosolic translation.^76^ More discriminating comparisons could use intact respiring mitochondria, membrane‐intact but respiration‐limited preparations, mtDNA‐depleted or fusion‐impaired donors, and matched cell‐free components. Because these manipulations can alter organelle fitness or clearance, donor quality, particle number, membrane integrity and early uptake should be characterised and matched where feasible. Washing, nuclease treatment and assays for free mtDNA or debris can help separate intact‐organelle effects from accompanying cargo.

Recipient‐side perturbations can test which processes are required after entry. Fusion, mitophagy and innate sensing can be tested separately while controlling for early uptake, donor and recipient viability, preparation quality and the recipient's basal mitochondrial function. Orthogonal reagents or washout controls help distinguish pathway effects from broader disruption of mitochondrial homeostasis. Under these controls, loss of sustained respiratory rescue after fusion blockade, despite preserved early cytokine responses, would support integration as a mechanism. If mitophagy inhibition preserves donor structure but removes benefit, productive processing is implicated. Disease‐relevant animal studies with cell‐type‐resolved donor tracing can then test whether the same requirement operates in vivo.

Time‐resolved analysis links mechanism to benefit duration. Early recovery establishes neither durable engraftment nor a purely signal‐mediated mechanism; bulk bioenergetic improvement leaves fusion status unresolved.^9^ Benefit may outlast donor material through recipient metabolic or immune reprogramming. Serial measurements should align donor‐specific mtDNA and organelle integrity with cytosolic mtDNA, cGAMP–STING activity, cytokines, bioenergetics, recipient biogenesis and disease outcomes. Attribution also requires perturbing the proposed mediator.

Sensing direction alone does not establish therapeutic value: activation may protect, inflame or facilitate immune evasion. Reduced cGAS–STING activity may reflect restored homeostasis and less endogenous mtDNA leakage despite earlier donor sensing.^12^, ^72^, ^75^ Persistent tracers, free donor mtDNA and debris obscure signal origins. Tests must separate donor‐organelle function, donor‐derived signalling and secondary recipient reprogramming.

The contribution that matters must last long enough to meet the therapeutic task.

## 4 WHEN MUST THE ORGANELLE PERSIST?

Acute injury may require transient rescue, chronic acquired disease sustained support, and heteroplasmic mtDNA disease durable correction. Each demands distinct evidence and product design.

Acute ischaemic injury may require time‐limited donor action. Myocardial, cerebral and renal ischaemia–reperfusion models^1^, ^2^, ^25^, ^27^, ^28^ offer salvage windows of hours to days. Transient respiration, calcium or redox buffering, or a protective recipient response may suffice. Later donor disappearance need not negate earlier benefit. Evidence should link exposure and uptake or cytosolic access to donor competence or mediator activity over the functional‐rescue interval.

Clinical implementation faces the same time constraint. Source‐tissue access, isolation and administration times restricted eligibility in the phase 1 cerebral‐ischaemia study.^4^ Preparation speed, integrity and delivery timing may therefore matter more than prolonged donor‐mtDNA persistence.

Chronic acquired disease requires sustained benefit, not necessarily lifelong retention of the original donor genome. Donor function, recipient adaptation or repeated replacement could maintain support as individual organelles disappear. Neurological and inflammatory models permit these routes but rarely distinguish them.^6^, ^29^, ^30^, ^31^, ^72^, ^77^ Evidence must span the claimed benefit; proposed redosing requires assessment of response and dosing interval.

Durable mtDNA correction requires more than transient bioenergetic support: donor wild‐type mtDNA must be retained and replicated to keep the pathogenic mtDNA heteroplasmy level below a functional threshold.^21^ Direct cytosolic transfer establishes that donor‐genome propagation is feasible.^37^, ^51^ In two patient‐derived fibroblast lines, a 2026 PN‐101 study found a short‐lived shift from mutant toward wild‐type mtDNA that required repeated treatment to maintain.^78^ That cellular response does not establish durable correction in vivo, which would require donor‐specific replication, sustained respiratory recovery and stability across cell division or tissue turnover.^53^

Nuclear‐gene mitochondrial disorders pose a different limit. In *NDUFS4*‐associated Leigh syndrome and *DGUOK*‐ or *TK2*‐related depletion syndromes,^79^, ^80^, ^81^ donor mtDNA cannot remove the causal nuclear lesion. Any sustained benefit must instead rely on donor‐organelle function, recipient adaptation or repeated replacement. PN‐101 increased mtDNA content and oxidative phosphorylation over 24–48 h in patient fibroblasts with *SSBP1*‐related depletion syndrome.^82^ This supports short‐term functional complementation; the requirements for sustained benefit remain unresolved.

Pronuclear transfer illustrates genome replacement in another setting. A clinical study showed that placing parental nuclear genomes in donor‐mitochondrial cytoplasm can reduce pathogenic‐mtDNA transmission risk.^83^ It precedes tissue disease and bypasses isolated organelles' uptake and sorting barriers, without testing somatic transplantation's post‐entry mechanisms.

Therapeutic duration and genetic purpose shape donor‐source selection alongside the delivery route. Autologous mitochondria may suit time‐limited rescue, but tissue‐specific mtDNA variants and heteroplasmy can leave accessible and diseased tissues with different mutation burdens.^71^ A lower‐burden autologous source is a testable option for mtDNA correction, not a default solution. Allogeneic or engineered mitochondria can provide a healthy genotype, but mtDNA haplotype, nuclear background, mitonuclear compatibility and repeated exposure require separate assessment.^62^, ^63^, ^64^, ^65^

Table 2 matches therapeutic tasks to evidence requirements; Figure 3 connects them to product design and recipient handling. These aims may overlap: donors could support early metabolism, initiate signalling and later contribute structurally.

**TABLE 2:** Indication‐specific post‐entry requirements and minimum evidence. The matrix links the therapeutic tasks in Section 4 to the contributions and evidence each requires, without ranking indications or prescribing one preferred fate.

Disease context and therapeutic task	Required post‐entry fate	Minimum evidence	Claim boundary
Acute ischaemic or reperfusion injury	Short‐term donor competence or productive processing within the injury window	Recipient‐cell uptake or cytosolic access; time‐matched donor integrity or mediator; early functional rescue; short‐term safety	No claim of durable engraftment, donor‐mtDNA persistence or genetic correction
Chronic acquired or degenerative disease	Sustained functional support through sustained donor function, recipient adaptation or reproducible replacement	Serial disease‐relevant function over the claimed benefit duration; redosing response and dosing intervals when intended; cumulative safety	No claim of permanent donor persistence or a universal mechanism
Heteroplasmic mtDNA disease	Donor‐mtDNA retention and replication sufficient for a stable shift below the pathogenic threshold	Donor‐specific replication; stable heteroplasmy shift; respiratory correction across cell division or tissue turnover	Donor‐sequence detection alone is insufficient for durable mtDNA correction
Nuclear‐gene mitochondrial disease	Sustained functional support through donor‐organelle function, recipient adaptation or repeated replacement	Duration of rescue and disease‐phenotype correction; donor‐specific respiratory contribution when claimed; dosing interval and redosing response when intended	Donor‐mtDNA persistence cannot correct the nuclear lesion

Abbreviation: mtDNA, mitochondrial DNA.

**FIGURE 3:**
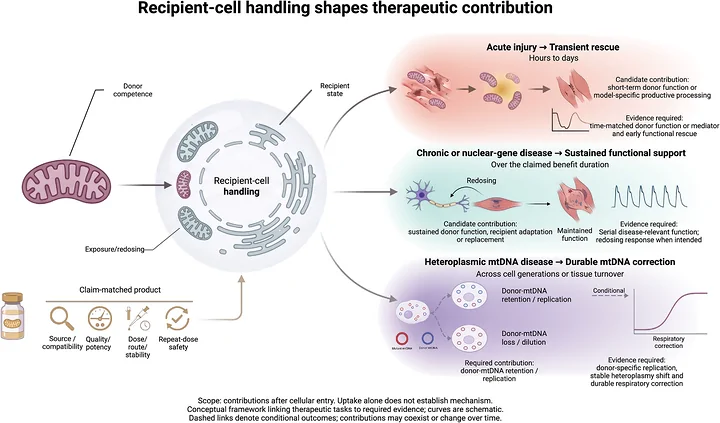
Recipient‐cell handling shapes therapeutic contribution. The framework links donor competence, recipient state and exposure or redosing to three therapeutic tasks after cellular entry. Product requirements cover source and compatibility, quality and potency, dose, route, stability and repeat‐dose safety. In acute injury, transient rescue over hours to days may involve short‐term donor function or productive processing in specific models. Testing this contribution requires linking donor function or a mediator to early rescue over the same interval. In chronic or nuclear‐gene disease, sustained support may depend on continuing donor function, recipient adaptation or replacement. Disease‐relevant function should be tracked over the claimed benefit duration, including dosing intervals when redosing is intended. For heteroplasmic mtDNA disease, durable mtDNA correction requires donor‐mtDNA retention and replication across cell generations or tissue turnover. Divergent lineages depict retention/replication and loss/dilution as alternative outcomes. Red and blue circles represent mutant and donor mtDNA, respectively. Donor‐specific replication, a stable heteroplasmy shift and sustained respiratory function must be assessed together to test the proposed correction. Arrows connect tasks to candidate contributions and required evidence. Dashed links indicate conditional outcomes; curves are schematic. Contributions may coexist or change over time, and uptake alone does not establish a mechanism. mtDNA, mitochondrial DNA.

## 5 TRANSLATING POST‐ENTRY FATE INTO A THERAPEUTIC PRODUCT

A mitochondrial product must be defined by its intended post‐entry contribution. Particle counts or protein content cannot establish how many organelles remain intact and respiration‐competent, reach the cytosol or persist.^5^, ^84^ Specifications must capture composition and intended function.

Donor source defines genotype and biological context; product quality describes the material at administration. Autologous material reduces sequence disparity, but it also carries biological variation related to patient age, comorbidity, tissue state and, in mitochondrial disease, the underlying causal defect.^71^, ^79^, ^80^, ^81^ Allogeneic or engineered sources can provide a more defined genotype and potentially greater scalability, with separate requirements for donor screening and compatibility assessment.^62^ Comparisons across donor sources reveal differences in mitochondrial properties and recipient responses.^85^, ^86^ Interpreting these differences requires recording tissue and cell state alongside mtDNA genotype, yield, membrane potential and respiratory competence.

Tissue origin adds compositional differences that donor–recipient relatedness does not capture. Mouse skeletal‐muscle mitochondria differ from liver mitochondria in cardiolipin composition and contain higher levels of selected respiratory‐chain and ATP‐synthase subunits.^87^ Lipidomic profiles also distinguish skeletal muscle from white and brown adipose mitochondria.^88^ Human tissues vary markedly in mtDNA copy number per diploid nuclear genome,^89^ which does not measure copies per isolated organelle. Skeletal‐muscle, adipose and placental preparations therefore warrant comparison of lipid composition, respiratory‐chain proteins and mtDNA content under matched isolation conditions before effects on fusion efficiency or immunogenicity can be established.

Isolation shapes organelle integrity and preparation composition. Depolarisation, uncoupling, membrane disruption, oxidative modification and mtDNA release are distinct forms of damage. Non‐mitochondrial debris and residual cellular material are separate compositional attributes. These states can coexist: depolarisation does not establish rupture, and respiration establishes neither mitochondrial enrichment nor absence of contaminants.

In a 2026 mouse xenotransplantation study, partially depolarised preparations retained activity. Progressive room‐temperature injury reduced respiration and therapeutic benefit, whereas fully disrupted material showed little activity.^90^ These findings link preparation state to function without establishing donor‐source equivalence or long‐term safety. Consensus guidance recommends reporting donor source, isolation method, particle size, free or vesicle‐enclosed status and post‐isolation modification.^14^ Filtration chemistry, isolation workflow and cryopreservation can alter composition, uptake, respiration and stability.^84^, ^91^ Release testing should combine mitochondrial enrichment and membrane integrity measurements with assessment of residual cells, free mtDNA, debris and route‐appropriate microbial contamination.

Potency assays must match the intended contribution; no assay captures every clinically relevant attribute. Transient‐rescue testing may assess membrane integrity, coupled respiration, ATP generation and stability over the administration window.^84^ Durable mtDNA correction requires genome integrity; network‐integration strategies also require fusion competence. Persistence and heteroplasmy emerge within recipients and cannot be established by release testing. Assays need validation against extended characterisation and intended function (Table 3).

**TABLE 3:** Translational requirements for a mitochondrial product. Product release and clinical assessment should reflect the intended post‐entry contribution. The listed domains are proposed minimum assessments, not universal regulatory specifications.

Product or clinical domain	Minimum assessment	Post‐entry question addressed	What it cannot establish alone
Source and compatibility	Donor tissue or cell type; autologous, allogeneic or engineered status; donor health; mtDNA and nuclear genotype; intended exposure duration	Genetic suitability, source consistency and compatibility questions	Organelle integrity, purity, potency or therapeutic benefit
Composition and purity	Mitochondrial enrichment; intact donor cells; nuclear DNA; free mtDNA; cellular debris; route‐appropriate microbial and endotoxin controls	Exposure to intact organelles versus accompanying cargo	Functional organelle contribution after entry
Integrity and potency	Membrane integrity and potential; coupled respiration; ATP‐generating capacity; oxidative modification; stability during administration	Short‐term donor competence and preparation‐associated integrity loss	Cytosolic access, structural integration or persistence
Dose, route and stability	Administered amount plus intact respiration‐competent fraction; harvest‐to‐dose time; storage; route; biodistribution	Recipient exposure and the expected duration of action	The active mechanism or a universal dose
Repeated dosing and immune safety	Serial cytokines, circulating free mtDNA, tissue injury and donor‐directed responses; mitonuclear compatibility across exposure durations	Cumulative sensing, clearance and compatibility risk	Long‐term safety inferred from single‐dose tolerance
Clinical outcome	Early organ function; donor‐specific tracking; persistence; redosing response; duration‐matched follow‐up	Whether the observed benefit matches the intended post‐entry fate	Mechanism from clinical improvement alone

Abbreviations: ATP, adenosine triphosphate; mtDNA, mitochondrial DNA.

Composition can alter both mechanism and safety. Free mtDNA, disrupted mitochondrial membranes, metabolites and non‐mitochondrial components may remain biologically active even when few intact organelles remain. Endosomal mtDNA can engage TLR9, whereas cytosolic mtDNA can activate cGAS–STING.^23^, ^67^, ^68^ Mitochondrial peptides and lipids provide additional danger‐associated signals.^11^, ^24^, ^69^ Damaged preparations may amplify inflammation, whereas intracellular processing of donor material can contribute to benefit in selected contexts.^12^, ^72^ A material balance across intact organelles, disrupted mitochondria and soluble cargo, followed where possible by testing matched fractions, can help distinguish these contributions.

Safety findings must be interpreted within the tested product and exposure conditions. One murine study detected no acute or chronic alloreactivity after single or serial administration of syngeneic or allogeneic mitochondria within the doses and routes examined.^65^ In the PN‐101 phase 1/2a study, nine adults with refractory idiopathic inflammatory myopathy received mitochondria derived from allogeneic umbilical‐cord mesenchymal stromal cells. After a single intravenous dose, transient treatment‐related reactions and preliminary activity were reported without severe adverse drug reactions.^70^ These human findings support early single‐dose feasibility, but do not establish source equivalence or safety under repeated or prolonged exposure. Expected donor‐mtDNA persistence or repeated systemic administration warrants longer surveillance of cytokine, humoral and cellular responses.^62^, ^63^, ^64^

Administration determines tissue exposure. Local injection concentrates the product; vascular delivery adds dilution, endothelial contact and off‐target distribution before parenchymal uptake.^25^, ^26^, ^27^, ^28^, ^29^ Report administered amount and, where possible, the intact, respiration‐competent fraction. Section 3.4’s dose plateaus could reflect saturated uptake, sufficient transient signalling or poorer preparations at higher concentrations. Assess biodistribution, clearance and organ‐specific safety by route; align redosing with donor function or recipient‐adaptation duration.

Human studies span different sources, routes and therapeutic settings. Autologous intramyocardial transplantation after paediatric ischaemia–reperfusion injury showed encouraging recovery signals in an early case series and a later retrospective comparison.^3^, ^92^ Intra‐arterial delivery during thrombectomy was feasible in four adults with acute cerebral ischaemia.^4^ PN‐101 adds single‐dose allogeneic treatment for inflammatory myopathy.^70^ A randomised study in 30 patients with ST‐elevation myocardial infarction tested intracoronary platelet‐derived mitochondria.^93^ In assisted reproduction, a 2026 sibling‐oocyte randomised trial of mitochondrial supplementation found accelerated cleavage timing without improving good‐quality embryo yield. Pregnancy and live‐birth comparisons were non‐randomised and did not demonstrate benefit.^94^ Table 4 summarises these designs and outcomes.

**TABLE 4:** Reported human studies of mitochondrial transplantation. Studies are described by indication, donor source, dose, route, design and follow‐up. Differences in these features limit cross‐study comparison; mechanistic coverage concerns post‐entry fate.

Clinical setting	Source, dose and route	Design and follow‐up	Main finding and evidence limit
Paediatric postoperative myocardial injury: early series (2017)	Autologous skeletal muscle; ∼10^8^ mitochondria in 1 mL, delivered as ten 100 µL intramyocardial injections in one procedure	Uncontrolled series, *n* = 5; serial echocardiography through day 10 and hospital outcomes^3^	Four patients separated from ECMO. No injection‐related arrhythmia or bleeding reported; efficacy and donor fate unresolved
Paediatric cardiogenic shock: retrospective comparison (2021)	Autologous rectus abdominis muscle; 1 mL respiration buffer (∼1 × 10^8^–1 × 10^9^ particles/mL). Direct epicardial/intramyocardial delivery: 10–20 injections, 50–100 µL and ∼1–5 × 10^7^ mitochondria each; median 1 day after revascularisation (range, 0.3–3 days)	Retrospective, 10 treated versus 14 controls; biomarkers from day −1 to day 7 around revascularisation^92^	ECMO separation without reinstitution within 1 week: 8/10 versus 4/14 (*p* = 0.02); median time to functional recovery after revascularisation: 2 versus 9 days (*p* = 0.02). Hospital and overall mortality did not differ significantly. Retrospective historical‐control comparison; donor fate unresolved
Acute cerebral ischaemia	Autologous skeletal muscle; single intra‐arterial infusion during thrombectomy. Estimated mean 1.335 × 10^6^ mitochondria^a^; protocol cap 7 × 10^6^ and ≤8 mL	Open‐label phase 1, four adults and 12 retrospectively matched controls; acute monitoring through one week^4^	Procedurally feasible; no monitored adverse events in treated patients. Small safety study; efficacy and donor fate unresolved
Refractory idiopathic inflammatory myopathy	Allogeneic umbilical‐cord‐MSC mitochondria (PN‐101); single intravenous dose. Administered protein dose: 300 µg (*n* = 4) or 600 µg (*n* = 5).	Open‐label multicentre phase 1/2a, nine treated adults; follow‐up at 4, 8, and 12 weeks^70^	Transient treatment‐related reactions; no severe adverse drug reactions. Uncontrolled improvement; donor fate and repeat‐dose safety unresolved.
ST‐elevation myocardial infarction	Platelet‐derived mitochondria^b^; 1.2 × 10^8^ in 10 mL respiration buffer. Intracoronary infusion via guiding catheter over 5–10 min after successful primary percutaneous coronary intervention; intervention within 24 h of ischaemia (mean, 5.053 h in the treatment group)	Randomised triple‐blind trial, 30 adults (15 per group); 40‐day follow‐up^93^	Exercise capacity: 12.5 versus 9.7 metabolic equivalents (*p* < .001). Left ventricular ejection fraction improved from baseline in both groups; no significant day‐40 between‐group difference (47.67% versus 45.67%; *p* = .90). No significant safety‐event differences at 24 h or 1 week. Small short‐term trial; donor fate not assessed
Recurrent assisted‐reproduction failure	Autologous bone‐marrow‐MSC mitochondria; ∼2 pL suspension co‐injected per allocated oocyte during ICSI. Organelle count/protein dose NR	Sibling‐oocyte randomisation: 151 patients, 1178 mature oocytes. Pregnancy/live‐birth assessment; reported offspring follow‐up to ages 2–6 years^94^	No improvement was demonstrated in good‐quality embryo yield or clinical outcomes. Pregnancy/live‐birth comparisons were non‐randomised. Reproductive delivery does not test somatic uptake

Abbreviations: ECMO, extracorporeal membrane oxygenation; ICSI, intracytoplasmic sperm injection; MSC, mesenchymal stromal cell; NR, not reported in the main article or supplementary tables examined.

^a^ Walker's value is the authors’ calculated transplant estimate (Table 3), not a direct organelle count. Protein amounts differ between Methods and Table 3; no mass‐to‐count conversion is imposed here. Counts, protein mass and suspension volume are not interchangeable.

^b^ Baharvand et al. described platelet‐derived mitochondria as autologous in the abstract but allogeneic in the main text and Methods. Platelets were supplied by the Iranian Blood Transfusion Organisation. Donor provenance therefore remains unresolved.

These studies support feasibility in specific settings, without establishing time‐resolved donor persistence or causal post‐entry mechanisms to Table 2’s standards. Table 2 guides mechanistic attribution, not eligibility for early trials. Acute‐rescue studies may prioritise product characterisation, short‐term safety and time‐matched organ function. Sustained‐support claims require serial function across the benefit duration and intended dosing intervals. Durable mtDNA correction requires tissue‐relevant retention, replication and heteroplasmy measurements.

Informative donor variants, clinically justified sampling and matched preclinical studies may help trace donor fate. Autologous sequence identity and limited repeat‐biopsy access constrain tracing. Circulating donor DNA indicates exposure, not necessarily target‐tissue intracellular persistence. Clinical benefit remains assessable when fate is unresolved; mechanistic claims must match measurements.

Each preparation must be traceable to a reproducible product. Link donor eligibility, source tissue, harvest‐to‐dose interval and isolation conditions^84^ to yield, release tests and process deviations. Rapid isolation enables near‐patient use but does not guarantee transport or storage stability.^91^ Autologous treatment requires validated processing and rapid assays; scalable allogeneic manufacture^95^ adds donor banking, batch comparability and longer‐term stability testing. Where possible, link each batch to recipient exposure, intended contribution and clinical outcome.

## 6 CONCLUSIONS AND PRIORITIES FOR THE FIELD

Delivery establishes exposure. Recipient‐cell handling shapes internalised mitochondria's contribution through compartmental sorting, network interaction, quality control and sensing. Extracellular effects offer another route to benefit beyond these post‐entry processes.

Transplantation studies establish cytosolic access, integration, donor‐mtDNA retention and productive processing in selected systems more clearly than their frequency, durability or therapeutic necessity. Time‐resolved fate mapping must separate cell association, cytosolic access, organelle integrity, network interaction, donor‐mtDNA persistence and function.

Causal tests must control donor quality, initial delivery, recipient basal mitochondrial function and network integrity when perturbing fusion, clearance or sensing. Rescue experiments and orthogonal approaches can strengthen inference. Donor‐specific and recipient‐specific measurements must cover the interval over which benefit is claimed. Transient rescue, sustained functional support and durable mtDNA correction require different evidence because they need not depend on the same donor fate or duration.

A donor mitochondrion may respire, buffer metabolism, contribute genetic material or release signals during processing. These roles may overlap or change. Persistence need not underlie every benefit; donor activity or the recipient response must meet the disease's needs for the required interval.

Linking product quality, recipient handling and outcomes can establish which post‐entry processes mediate benefit and guide experimental and clinical design.

The central question is no longer simply how many mitochondria can be delivered. It is what recipient cells permit, reject or transform after donor mitochondria arrive—and which of those outcomes is responsible for benefit.

## AUTHOR CONTRIBUTIONS

Zhi‐Yuan Zhang and Bo‐Quan Qin wrote the manuscript and prepared Figures 1, 2, 3 and Box 1. Shi‐Zhou Wu and Hui Zhang revised the manuscript and figures. All authors reviewed the manuscript.

## CONFLICT OF INTEREST STATEMENT

The authors declare no conflict of interest.

## CONSENT

Not applicable.

## DECLARATION OF GENERATIVE AI AND AI‐ASSISTED TECHNOLOGIES IN THE WRITING PROCESS

In preparing the text, the authors used ChatGPT for language editing, text organisation and reference checking. The authors reviewed and edited the output and take responsibility for the publication.

## ETHICS STATEMENT

This narrative Review reports no new studies involving human participants or animals.

## Data Availability

No new data were generated or analysed for this review.
